# Hypogonadotropic Hypogonadism and Kleefstra Syndrome due to a Pathogenic Variant in the* EHMT1* Gene: An Underrecognized Association

**DOI:** 10.1155/2018/4283267

**Published:** 2018-10-02

**Authors:** Ana Patricia Torga, Juanita Hodax, Mari Mori, Jennifer Schwab, Jose Bernardo Quintos

**Affiliations:** ^1^Summer Intern, Division of Pediatric Endocrinology and Diabetes, Rhode Island Hospital/Hasbro Children's Hospital, 111 Plain St, 3rd Floor, Providence, RI 02903, USA; ^2^Division of Pediatric Endocrinology and Diabetes, Rhode Island Hospital/Hasbro Children's Hospital, The Warren Alpert Medical School of Brown University, 111 Plain St, 3rd Floor, Providence, RI 02903, USA; ^3^Division of Human Genetics, Hasbro Children's Hospital, The Warren Alpert Medical School of Brown University, 2 Dudley Street, Suite 460, Providence, RI 02903, USA

## Abstract

Kleefstra syndrome is a genetic condition characterized by intellectual disability, childhood hypotonia, and facial dysmorphisms. Genital anomalies such as micropenis, cryptorchidism, and hypospadias have been reported in 30-40% of males diagnosed with the disease. However, endocrinological investigations have been limited. We describe a case of an adolescent male with Kleefstra syndrome due to a pathogenic variant in the* EHMT1* gene whose workup for isolated micropenis is suggestive of a partial hypogonadotropic hypogonadism. A possible endocrine mechanism of the genital anomaly associated with Kleefstra syndrome is discussed.

## 1. Introduction

Kleefstra syndrome is characterized by intellectual disability and childhood hypotonia with associated distinctive facial dysmorphisms. A heterozygous microdeletion at chromosome 9q34.3 overlapping the euchromatin histone methyltransferase 1 (*EHMT1*) gene accounts for more than 75% of cases, and the remainder are associated with a heterozygous intragenic pathogenic variant in the* EHMT1 gene* [1, 2]. Prevalence data is limited but it has been estimated at 1 per 200,000 individuals diagnosed with intellectual disability [3]. Typical phenotypes highly associated with the syndrome include heart defects, seizures, obesity, eye abnormalities, behavioral problems, and genital abnormalities. Penetrance is likely 100% with variable expressivity.

Genital anomalies including micropenis, cryptorchidism, and hypospadias have been reported in 30-40% of male patients with Kleefstra syndrome [4]. The mechanisms of genital anomalies among these patients have yet to be established and there are no reports of endocrinological investigations of these patients.

We describe a case of an adolescent male diagnosed with Kleefstra syndrome due to a de novo pathogenic variant c.2712+1G>A in the* EHMT1* gene and isolated micropenis. The boy's clinical presentation, workup, management, and possible endocrine mechanisms underlying his genital anomaly are discussed.

## 2. Case Presentation

The patient is an 11-year-old boy with Kleefstra syndrome whom we first evaluated in the endocrine clinic at 8 years of age for obesity to rule out Prader-Willi Syndrome. The patient is the male child of nonconsanguineous Guatemalan parents and was born at 41 weeks of gestation by spontaneous vaginal delivery to a 23-year-old, gravida 2, para 1 mother. The pregnancy was not complicated by any exposure to viral infection or medications. His siblings and both parents are healthy with no family history of miscarriages, stillbirths, congenital abnormalities, or learning difficulties. He was reportedly well until the 19th day of life when he presented with projectile vomiting and was diagnosed with pyloric stenosis. Surgery was uncomplicated; however, he had recurrent surgical site infections which required multiple readmissions.

In the interim, parents reported that he was able to walk at 3 years of age and had his first meaningful word (“Papa”) at 16 months. He attended special education classes and received speech, occupational, and physical therapy to address his developmental delays. He had recurrent acute otitis media managed with bilateral myringotomy. Audiologic evaluation also showed conductive hearing loss.

**Figure 1:**
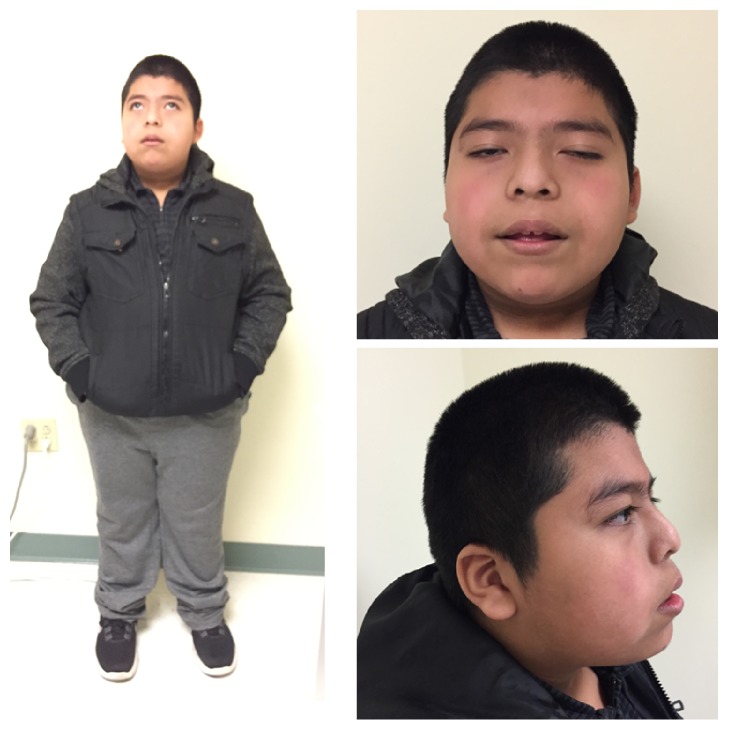
Index patient showing facial dysmorphism (prominent eyebrow, low set ears, midfacial retrusion, and mild prognathism).

He was evaluated by endocrinology for the first time at age 8 years and 8 months. He was referred by his pediatrician for evaluation of obesity and hyperphagia which raised concern for possible Prader-Willi Syndrome. His height was 134.1 cm (64th percentile), weight 63.5 kg (>99th percentile), and BMI 35.31 kg/m2. Examination was remarkable for facial dysmorphisms (prominent eyebrows, low set ears, midfacial retrusion, and mild prognathism) (see Figure 1) and a genital exam that showed a micropenis. He was prepubertal with 3 cc testicles bilaterally, stretched penile length measured at 3 cm (-2.5 SD for age is 2.8 cm; mean is 6.3 cm), and no hypospadias. Laboratory tests showed LH <0.005 mIU/ml (normal 0.02-0.3), FSH 0.184 mIU/ml (normal 0.26-3), and testosterone 9 ng/dL (normal 2.5-10) (Table 1). Brain MRI was normal.

Due to the low gonadotropins associated with isolated micropenis, treatment was initiated via intramuscular testosterone cypionate injection of 50 mg given once a month for 3 months at the age of 9 years and 9 months. He had a normal response to testosterone injections with an improvement of stretched penile length to 5.5 cm (normal 6.3 ± 1.0 cm) after 4 doses. There were no noted adverse reactions to testosterone injections such as acne, fluid retention, decreased testicular size, or mood swings.

At 10 years and 9 months, GnRH agonist stimulation testing showed an LH-predominant response with peak LH of 11 mIU/mL and peak FSH of 4.3 mIU/mL at 24 hours. Testosterone rose from 48 ng/dL (normal <7-130 ng/dL) at baseline to 132 ng/dL at 48 hours (Table 2). hCG stimulation testing was done 1 month later for Leydig cell function assessment.  Table 3 shows that there was adequate testosterone biosynthesis (testosterone 300 ng/dL at 24 hours after the last dose of hCG) and no evidence of 5-alpha reductase deficiency (T:DHT 21.4; normal T:DHT <35) after the hCG stimulation test.

Genetics referral was initially made at 1 year of age due to global developmental delay, nontypical dysmorphic facial features, and the history of hypertrophic pyloric stenosis. There was no history of hypotonia, feeding difficulties, or seizures. Karyotype showed normal male (46, XY). Fragile X testing was normal. DNA oligonucleotide microarray study revealed a likely benign maternally inherited 563 kb duplication at 1p22.3. Rett syndrome, although rare in males, was ruled out at the age of 4 by sequencing and deletion/duplication analysis of the MECP2 gene. At 9 years of age, methylation study for Prader-Willi critical region was negative. Whole exome sequencing (WES) revealed a heterozygous de novo pathogenic variant c.2712+1G>A in the* EHMT1* gene, which led to a diagnosis of Kleefstra syndrome. Mitochondrial DNA was sequenced as part of the WES with normal result. Developmental delays, dysmorphic facies, genital abnormalities, obesity, hearing loss, and recurrent infections are consistent with the diagnosis (see Table 4). Screening tests for other associated phenotypic presentations were done. Echocardiogram and renal ultrasound were negative.

Follow-up exam at 11 years of age showed Tanner stage 3 pubic hair, testicular volume of 6 cc bilaterally, and stretched penile length 6 cm (normal 6.4 ± 1.1 cm). Parents deny any new behavioral changes, sleep disturbances, or seizures. He continues to follow up in the endocrine clinic for monitoring of pubertal progression and growth velocity.

## 3. Discussion

We report the case of an 11-year-old male with Kleefstra syndrome, isolated micropenis, and possible hypogonadotropic hypogonadism. We propose partial hypogonadotropic hypogonadism as an underlying mechanism of micropenis in association with the genetic syndrome. This case report provides an extensive endocrinology workup to further elucidate this mechanism.

Partial hypogonadotropic hypogonadism is known to cause incomplete pubertal development in both boys and girls. Male patients with partial hypogonadotropic hypogonadism can present with micropenis, cryptorchidism, stalled pubertal progression, or hypogonadism with normal testicular volume (Fertile Eunuch Syndrome) [5]. Female patients can present with incomplete breast development and primary amenorrhea [6].

The initial labs of our patient showed undetectable LH and FSH levels suggestive of hypogonadotropic hypogonadism. After testosterone replacement therapy, a normal hypothalamic-pituitary-gonadal axis response on GnRH agonist and hCG stimulation tests was noted. This can be explained by a partial hypogonadotropic hypogonadism. Normalization of gonadotropin levels after treatment has been reported in 10% of males with idiopathic hypogonadotropic hypogonadism in a retrospective study done to reevaluate the need for lifelong hormonal therapy [7]. There are also reports of spontaneous reversal of idiopathic hypogonadotropic hypogonadism associated with the Fertile Eunuch variant [5]. This reflects the plasticity of the reproductive neuroendocrine system. It also highlights the importance of conducting an endocrine evaluation in patients presenting with potentially treatable genital anomalies including those associated with genetic syndromes such as Kleefstra syndrome.

Kleefstra syndrome is a recently identified cause of intellectual disability with associated childhood hypotonia and distinctive facial dysmorphism. More than 75% of the cases reported in the literature have been found to have a subtelomeric deletion at chromosome 9q34.3 while the remaining cases are due to a heterozygous pathogenic variant of the EHMT1 gene [4]. Similar phenotypic presentations such as cardiac defects, obesity, and seizures have been reported for the two genotypes.

Molecular testing confirmed a de novo heterozygous pathogenic variant in the* EHMT1* gene diagnostic of Kleefstra syndrome. The variant destroys a canonical splice donor site and likely leads to a truncated protein [8].* EHMT1* is expressed in multiple tissues including the brain, eyes, male embryonic germ cells, epididymis, ovary, heart, and aorta [9]. In the brain, it is expressed in the hypothalamus and the pituitary gland showing that it can potentially play a role in gonadotropin secretion.

Only male patients present with genital anomalies such as micropenis, cryptorchidism, and hypospadias but these have been reported in both genotypes (62.5% with microdeletions, 33% with* EHMT1* gene mutation). Case series on Kleefstra syndrome [1, 4, 10] do not report the endocrine evaluation of these patients presenting with associated genital anomalies limiting the available pool of knowledge to identify its underlying mechanism. Micropenis etiology can be classified as primary hypogonadism, insufficient testosterone secretion (hypogonadotropic hypogonadism), testosterone activation defect, developmental, or idiopathic [11]. Chromosome 9q34.3 overlaps the nasal embryonic LH-releasing hormone factor (NELF) gene which is associated with some patients with autosomal dominant idiopathic hypogonadotropic hypogonadism [12]. It can be speculated that loss of function in the gene underlies hypogonadotropic hypogonadism in 9q34.3 deletion-related Kleefstra syndrome. However, in patients with* EHMT1* gene pathogenic variants such as our patient the mechanism remains unclear.

Assessment of the serum gonadotropins, pituitary hormones, testosterone, and its derivatives is a practical starting point to determine the level at which the hypothalamic-pituitary-gonadal axis is affected [13]. Ideally, the GnRH agonist stimulation testing should have been done prior to testosterone therapy. However, both LH and FSH were quite low for age at onset, which makes partial hypogonadotropic hypogonadism a strong differential for micropenis in this patient in the setting of Kleefstra syndrome.

Partial hypogonadotropic hypogonadism could be a potential cause of isolated micropenis in some patients with Kleefstra syndrome. This case is a reminder that genital anomalies associated with Kleefstra syndrome due to an intragenic variant in the* EHMT1* gene warrant investigation of underlying neuroendocrine imbalances that may cause genital anomalies and affect the progression of puberty, especially during adolescence. Continued monitoring of pubertal progression should be done in patients treated with hormone-replacement therapy for micropenis. There is still a possibility of stalled pubertal progression in patients with partial hypogonadotropic hypogonadism. Findings of continuous testicular growth and increase in serum testosterone in the absence of treatment are strong indicators of a normally functioning hypothalamic-pituitary-gonadal axis [7]. Maintenance of a normal hormonal status in patients with partial hypogonadotropic hypogonadism is expected to have positive impacts on other aspects of development (e.g., achievement of genetic potential for height, lean muscle mass, bone mineralization, metabolism, mood, and cognitive function) [14]. Further studies are needed to elucidate the mechanism of the association of hypogonadotropic hypogonadism with pathogenic variants in the* EHMT1* gene.

## Figures and Tables

**Table 1:** Summary of initial endocrine hormones done at the age of 9 years and 4 months.

LH (0.02 - 0.3)	<0.005 mIU/mL
FSH (0.26-3)	0.184 mIU/mL
Testosterone (2.5-10)	9 ng/dL
Free T4 (0.8-1.8)	0.81 ng/dL
AM Cortisol (>10)	10.5 mcg/dl

**Table 2:** GnRH agonist stimulation test (leuprolide acetate) done at the age of 10 years and 9 months using 20 mcg/kg/subcutaneous dose.

	**Baseline**	**60 min**	**120 min**	**24 hrs**
**LH** (mIU/mL)	1.7	9.2	10	11
**FSH** (mIU/mL)	2.3	3.4	3.7	4.3
**Testosterone** (ng/dL)	48	-	-	132

**Table 3:** hCG stimulation test done at the age of 10 years and 10 months with 5000-unit hCG once a day for 3 days.

	**Baseline (Day 1)**	**Day 3**	**Day 6**

**Testosterone** (ng/dL)	43	300	298
**DHT** (ng/dL)	4.6	14	15
**T:DHT** (<35)	9.3	21.4	19.1
**Androstenedione** (ng/dL)	85	105	66
**T:Androstenedione** (>0.8)	0.5	2.9	4.5

**Table 4:** Summary of clinical features seen in 21 patients [4] with *EHMT1* mutations and in the index patient.

**Clinical Features**	%** in 21 patients**	**Index Patient**
Short Stature	17	-
Overweight (BMI>25)	42	+
Developmental Delay/Intellectual Disability	100	+
Heart defect	43	-
Genital anomaly (in males)	43	+
Renal anomaly	14	-
Recurrent Infections	64	+
Hearing deficit	24	+
Gastro-esophageal reflux	14	-
Epilepsy	24	-
Behavioral/psychiatric problems	75	-
Anomalies on brain imaging	63	-
Tracheomalacia	5	-
Umbilical/inguinal hernia	10	-
Anal atresia	5	-
Musculoskeletal anomaly	19	-
Respiratory complications	5	-
